# Antibody Responses to SARS-CoV-2 Following an Outbreak Among Marine Recruits With Asymptomatic or Mild Infection

**DOI:** 10.3389/fimmu.2021.681586

**Published:** 2021-06-09

**Authors:** Irene Ramos, Carl Goforth, Alessandra Soares-Schanoski, Dawn L. Weir, Emily C. Samuels, Shreshta Phogat, Michelle Meyer, Kai Huang, Colette A. Pietzsch, Yongchao Ge, Brian L. Pike, James Regeimbal, Mark P. Simons, Michael S. Termini, Sindhu Vangeti, Nada Marjanovic, Stephen Lizewski, Rhonda Lizewski, Mary-Catherine George, Venugopalan D. Nair, Gregory R. Smith, Weiguang Mao, Maria Chikina, Christopher C. Broder, Eric D. Laing, Alexander Bukreyev, Stuart C. Sealfon, Andrew G. Letizia

**Affiliations:** ^1^ Department of Neurology, Icahn School of Medicine at Mount Sinai, New York, NY, United States; ^2^ Naval Medical Research Center, Silver Spring, MD, United States; ^3^ Department of Microbiology and Immunology, Uniformed Services University of the Health Sciences, Bethesda, MD, United States; ^4^ Henry M. Jackson Foundation for the Advancement of Military Medicine, Inc., Bethesda, MD, United States; ^5^ Department of Pathology, University of Texas Medical Branch, Galveston, TX, United States; ^6^ Galveston National Laboratory, University of Texas Medical Branch, Galveston, TX, United States; ^7^ Directorate for Public Health, Navy Medicine Readiness and Training Command Beaufort, Beaufort, SC, United States; ^8^ Department of Parasitology, Naval Medical Research Unit 6, Lima, Peru; ^9^ Department of Bacteriology, Naval Medical Research Unit 6, Lima, Peru; ^10^ Department of Computational and Systems Biology, School of Medicine, University of Pittsburgh, Pittsburgh, PA, United States; ^11^ Department of Microbiology & Immunology, University of Texas Medical Branch, Galveston, TX, United States

**Keywords:** SARS-COV-2, antibodies, outbreak, young adults, COVID-19

## Abstract

We investigated serological responses following a SARS-CoV-2 outbreak in spring 2020 on a US Marine recruit training base. 147 participants that were isolated during an outbreak of respiratory illness were enrolled in this study, with visits approximately 6 and 10 weeks post-outbreak (PO). This cohort is comprised of young healthy adults, ages 18-26, with a high rate of asymptomatic infection or mild symptoms, and therefore differs from previously reported longitudinal studies on humoral responses to SARS-CoV-2, which often focus on more diverse age populations and worse clinical presentation. 80.9% (119/147) of the participants presented with circulating IgG antibodies against SARS-CoV-2 spike (S) receptor-binding domain (RBD) at 6 weeks PO, of whom 97.3% (111/114) remained positive, with significantly decreased levels, at 10 weeks PO. Neutralizing activity was detected in all sera from SARS-CoV-2 IgG positive participants tested (n=38) at 6 and 10 weeks PO, without significant loss between time points. IgG and IgA antibodies against SARS-CoV-2 RBD, S1, S2, and the nucleocapsid (N) protein, as well neutralization activity, were generally comparable between those participants that had asymptomatic infection or mild disease. A multiplex assay including S proteins from SARS-CoV-2 and related zoonotic and human endemic betacoronaviruses revealed a positive correlation for polyclonal cross-reactivity to S after SARS-CoV-2 infection. Overall, young adults that experienced asymptomatic or mild SARS-CoV-2 infection developed comparable humoral responses, with no decrease in neutralizing activity at least up to 10 weeks after infection.

## Introduction

Humoral immune responses to severe acute respiratory syndrome coronavirus 2 (SARS-CoV-2) are induced within a few days after infection in most infected individuals (1–4). Neutralizing antibodies to SARS-CoV-2 are stable for at least 5-8 months after infection (5–7). A high proportion of SARS-CoV-2 infections, particularly in young adults, occurs in the absence of symptoms (8, 9). Although individuals with asymptomatic infections develop SARS-CoV-2 antibodies, it has been reported that the magnitude of their response is less than for symptomatic individuals (10, 11). Given the elevated proportion of asymptomatic and mild infections in young populations, and their high potential for transmission (12), characterizing the duration and efficacy of their immune response is critical to establishing efficient public health measures for military training as well as local communities.

In the context of military settings, infectious diseases such as SARS-CoV-2 can lead to loss of workdays for young adults, degrading training and readiness (13–16). Additionally, the normal countermeasures to control outbreaks, such as quarantine and reinforced hygiene requirements, may be insufficient for Coronavirus Disease 2019 (COVID-19) prevention (12). Between March 15 and April 15, 2020, multiple recruits at Marine Corp Recruit Depot Parris Island (MCRDPI) presented with acute respiratory symptoms including cough, fever, muscle aches, shortness of breath, and sore throat. A total of 147 participants that were identified as infected with, or exposed to, SARS-CoV-2, and were isolated from the rest of the Marine recruits in training, were included in this study. Here, we characterize the serological responses to SARS-CoV-2 and other betacoronaviruses during an early outbreak in US Marine recruits over a 28 day period, approximately 6 and 10 weeks after SARS-CoV-2 exposure, and assess the relationship with disease manifestation among a Marine recruit training population.

## Methods

### Cohort and Data Collection

147 participants that were isolated during an outbreak of respiratory illness between March 15 and April 15 (outbreak window), 2020, were enrolled in this study. The criteria for inclusion in this study were: i) being 18 years or older at the time of the study enrollment and ii) potential or confirmed exposure during the outbreak window, defined as ii.a) suspected COVID-19 by having met clinical case definition according to Centers for Disease Control and Prevention (17), or ii.b) having confirmed SARS-CoV-2 infection using BioFire COVID-19 test (BioFire Defense, Salt Lake City, UT, USA) as part of medical care during the outbreak window, or ii.c) being in contact with suspected or confirmed SARS-CoV-2 positive individuals and were deemed persons under investigation. As part of the protocol, the investigators reviewed the electronic medical records for the consented participants. Enrollment in the study was approximately 6 weeks (4-8 weeks range) post-outbreak (PO), and a subsequent follow-up occurred 4 weeks later (10 weeks PO, 8-12 weeks range). At both time points, the participants were asked to complete a short questionnaire to self-report symptoms of respiratory infection and contact with anyone with respiratory symptoms. Demographic information, such as age, sex, ethnicity, and smoking history was collected at the initial enrollment. Signs and symptoms included in the questionnaires were: fever (>100.4), subjective fever, chills, muscle aches, fatigue, runny nose, sore throat, cough, shortness of breath, nausea or vomiting, headache, taste or smell decreased ability, abdominal pain, diarrheal, or others.

### Collection of Biological Specimens

At each time point (6 and 10 weeks PO), blood was collected using serum separator tubes (SST) and serum separated by centrifugation (1500 x g for 10 min). Aliquots of serum were frozen at -80°C. Nares swab collection was performed in the field at 10 week PO for quantitative polymerase chain reaction (qPCR) testing by Lab24Inc (Boca Raton, FL) using the FDA Emergency Use Authorized (EUA) Thermofisher TaqPath™ COVID-19 Combo Kit (Thermo Fisher Scientific, Waltham, MA) (12).

### Enzyme-Linked Immunosorbent Assay (ELISA) for Evaluation of SARS-CoV-2 RBD Specific IgG and IgM Titers

IgG and IgM SARS-CoV-2 specific antibodies in serum were evaluated using an enzyme-linked immunosorbent assay (ELISA) as previously described (12). Briefly, serum samples were heat inactivated at 56°C for 1 h. ELISA 384-well Immulon 4 HBX plates (Thermo Fisher Scientific) were coated overnight at 4°C with recombinant His-tagged SARS-CoV-2 receptor binding domain (RBD) (SinoBiological, Beijing, China) at a concentration of 2 µg/ml in phosphate-buffered saline (PBS). Plates were washed three times with 0.1% Tween-20 (Thermo Fisher Scientific) PBS (PBS-T) using an automated ELISA plate washer (Aquamax 4000, Molecular devices, San Jose, CA), and blocked for 1 h at room temperature (RT) with 3% milk PBS-T. Blocking solution was removed, and 6 serial dilutions of serum (3-fold dilutions starting at 1:50, prepared in 1% milk PBS-T) were dispensed in the wells. At least 2 positive controls (sera with qualified SARS-CoV-2 RBD reactive IgG and IgM), 8 negative controls (sera collected before July 2019, BioChemed Services, Winchester, VA) and 4 blanks (no sera) were included in every plate. Plates were incubated for 2 h at room temperature, and then washed 3 times with PBS-T. Next, peroxidase conjugated goat F(ab’)2 anti-human IgG (Abcam, Cambridge, UK) or goat anti-human IgM mu chain (Abcam) were added at a dilution of 1:5000 in 1% milk PBS-T, and plates were incubated for 1 h at RT. Plates were washed 6 times with PBS-T, developed using SIGMAFAST™ OPD (Sigma-Aldrich, St. Louis, MO), and the reaction was stopped after 10 min with 3M HCl (Thermo Fisher Scientific). Optical density (OD) at 492 nm was measured using a microplate reader (SpectramaxM2, Molecular Devices). Each dilution was considered positive when its OD 492 nm value was higher than the average of the negative controls plus 3 times their standard deviation (SD) at the correspondent dilution or higher than OD 492nm of 0.15. Samples were considered positive for RBD reactive IgG or IgM when positive results were obtained for at least 2 consecutive dilutions. Area under the curve (AUC) values were calculated using the six dilutions assayed for RBD IgG and IgM antibodies.

### Luminex xMAP-Based Multiplex Assays for Serology of SARS-CoV-2 Antigens and S Proteins From Zoonotic and Human Endemic Betacoronaviruses

Evaluation of IgG and IgA antibody levels to spike (S) glycoprotein antigens, S1 subunit, S2 subunit, RBD, and to nucleocapsid (N) protein of SARS-CoV-2 was performed with the MILLIPLEX^®^ Multiplex Immunoassays SARS-CoV-2 Antigen Panel 1 IgG and SARS-CoV-2 Antigen Panel 1 IgA (Millipore). Prefusion stabilized trimeric S proteins of SARS-CoV-2, SARS-CoV, MERS-CoV, and seasonal human coronaviruses (HCoV) HCoV-HKU1 and HCoV-OC43, as well as SARS-CoV-2 RBD were utilized in a betacoronavirus multiplex microsphere-based immunoassay (MMIA) to detect IgG as previously described (18–20). Briefly, serum samples were diluted 1:400 in PBS and tested in technical duplicates. After incubation, S protein-bead captured IgG were detected with biotinylated cross-absorbed anti-human IgG. Lastly, streptavidin-phycoerythin was added to each well and antigen-antibody complexes were analyzed on Bio-Plex 200 HTF multiplexing systems (Bio-Rad).

### Neutralization Assays

Studies involving infectious SARS-CoV-2 were performed at the Galveston National Laboratory in biosafety level 4 (BSL4) conditions. Vero E6 cells were seeded in 96-well optical black plates in Minimum Essential Medium (Thermo Fisher Scientific; Cat No. 11095080) containing 10% fetal bovine serum (FBS) and 0.05 g/L Gentamicin sulfate and incubated overnight at 37°C with 5% CO_2_. Serum samples, heat-inactivated at 56°C for 30 min, were two-fold serially diluted from an initial dilution of 1:10 in FBS free media (Minimum Essential Medium containing 25 mM HEPES and 0.05 g/L Gentamicin sulfate) and incubated with an equal volume of mNeonGreen SARS-CoV-2 (21) for 1 hour at 37°C at a final concentration of 200 PFU in humidified 5% CO_2_. Virus-serum mixtures were then added to Vero-E6 monolayers in 96 well optical black plates and incubated at 37°C. Plates were read using the BioTek Cytation 5 plate reader (EX 485 nm, EM 528 nm) at 24 h post-infection. Following background signal correction, neutralization of virus infection in the presence of serum dilutions was calculated as a percent of virus infection without serum. Curves of percent neutralization were plotted with Prism version 9 (**Supplementary Figure 1**) and a 4-parameter logistic regression method was used to calculate the serum dilution at which 50% of virus was neutralized (half-maximal inhibitory serum dilution; ID_50_).

### Statistics

Statistical analysis was performed with the Prism 9 software, RStudio (version 1.3.1093) and R (version 4.0.2). For serology assays, paired two or three way-ANOVA was performed, followed by multiple comparisons using Benjamini-Hochberg method, with a desired false discovery rate (FDR) of less than 0.05. Correlations were evaluated using the Pearson method. Distribution of ethnicity, race and sex among study groups was assessed with a Pearson’s Chi-squared test followed post-hoc analysis based on residuals, adjusted using the Bonferroni method.

### Study Approval

This study was approved by the Naval Medical Research Center (NMRC) institutional review board (IRB), protocol number NMRC.2020.0006, in compliance with all applicable U.S. federal regulations governing the protection of human subjects. Research performed at Icahn School of Medicine at Mount Sinai (ISMMS) as part of this study was reviewed by the ISMMS Program for Protection of Human Subjects and the Naval Information Warfare Center Pacific (NIWC Pacific) Human Research Protection Program (HRPP) and received non-human subjects (NHS) determination. All participants provided written informed consent.

## Results

### Cohort Characteristics and Classification According to Serology and Symptoms

The outbreak study population consisted of 147 participants (74.8% male, mean age 19.8 years [SD 1.69]). The participants identified their race as either White (69.4%), Black (12.9%), Asian (2.0%), American Indian/Alaska Native (2.0%), Native Hawaiian/Other Pacific Islands (0.6%) or non-specified (10.2%), and their ethnicity as Hispanic (26.5%), Non-Hispanic (38.1%) or non-specified (35.4%). The five most identified signs and symptoms at the time of COVID-19 diagnosis were active cough (n=81; 38.2%), sore throat (n=67; 31.6%), rhinitis (n=50; 23.6%), fever over 100.4°F (n=33; 15.6%), and headache (n=28; 13.2%). 15 participants were diagnosed as SARS-CoV-2 positive using the BioFire COVID-19 test at the time of the outbreak. SARS-CoV-2 RBD IgG titers were detected in 119/147 (80.9%) participants at the 6 week PO time point by ELISA. The serology for 114 of the participants who were positive at 6 weeks PO was also performed at 10 weeks PO, and 111 (97.3%) of them still showed presence of RBD IgG specific antibodies. Out of the 28 IgG negative participants at 6 weeks, 22 were tested again at 10 weeks, and 27 of them remained negative, while one participant became positive during this time. All 15 participants that have had confirmed SARS-CoV-2 infection by BioFire PCR testing also had IgG antibodies at 6 and 10 weeks PO. A total of 67.3% (99/147) of the participants presented with SARS-CoV-2 RBD specific IgM antibodies at 6 weeks PO, and this percentage was reduced to 43.1% (59/137) 10 weeks PO. None of the participants required hospitalization, as all were treated as outpatients. As expected, among the 136 participants tested at 10 weeks PO for active SARS-CoV-2 infection by swab PCR, all were negative.

To better understand the relationship between symptomatic infection and serological responses, we classified these participants into four groups based on serological evidence of previous infection and on the symptoms documented during the outbreak, obtained by review of the electronic medical record. To accommodate the symptoms heterogenicity of this group for downstream analysis, the following categories were established (**Table 1**): i) Asymptomatic (As): IgG RBD positive, 0 symptoms; ii) Mild presenting with low number of symptoms (MiL): IgG RBD positive, 1-3 symptoms, none of them fever or shortness of breath; iii) Mild presenting with higher number of symptoms (MiH): IgG RBD positive, more than 3 symptoms, or less than 3 but fever or shortness of breath was present; iv) Negative (Neg): IgG RBD negative. Some of the participants in the Neg group (12/27) presented mild symptoms (**Table 1**), probably due to infections other than SARS-CoV-2. This high frequency of symptoms in the Neg group can be explained by the fact that the presence of respiratory symptoms was part of the inclusion criteria. Alternatively, those Neg subjects could have been potentially infected by SARS-CoV-2, but did not generate or maintain detectable specific antibodies at 6 weeks PO. Ethnicity and race distribution was balanced across the participants in the As, MiL, MiH and Neg groups (**Table 2**, p-value = 0.2079 and 0.8076 for ethnicity and race, respectively). Male participants were more represented than female participants in the As group as compared with the rest of the groups (post-hoc adjusted p-value = 0.0165).

**Table 1:** Classification of participants based on serology and symptoms during the time period of the outbreak.

Category	IgG RBD	Number of symptoms (03/15/20-04/15/20)	Symptoms reported (in different numbers and combinations)	n
Asymptomatic (As)	Positive	0	none	55
Mild with low number of symptoms (MiL)	Positive	1-3, none of them fever or shortness of breath	Runny nose; Sore throat; Cough (new onset or worsening of chronic cough); Subjective fever (felt feverish); Headache; Chills; Nausea or vomiting	39
Mild with high number of symptoms (MiH)	Positive	>3 symptoms,	Runny nose; Sore throat; Cough (new onset or worsening of chronic cough); Chills; Subjective fever (felt feverish); Nausea or vomiting; Diarrhea; Abdominal Pain;	25
		or <3 but presented with fever or shortness of breath	Headache; Shortness of breath; Fever >100.4; Muscle aches; Fatigue; Taste or smell Decreased ability	
Negative serology (Neg)	Negative	0-4	Runny nose; Sore throat; Cough (new onset or worsening of chronic cough); Subjective fever (felt feverish); Headache; Chills; Nausea or vomiting	28
		(12/28 participants with 1-4 symptoms)		

**Table 2:** Contingency table showing the distribution of ethnicity, race and sex in As, MiL, MiH and Neg groups.

Ethnicity	As	MiL	MiH	Neg
Hispanic	13 (23.6%)	9 (23.1%)	12 (48%)	5 (17.9%)
Non-Hispanic	21 (38.2%)	14 (35.9%)	8 (32%)	13 (46.4%)
Non-specified	21 (38.2%)	16 (41%)	5 (20%)	10 (37.7%)
Chi-squared p-value = 0.2079				
**Race**	**As**	**MiL**	**MiH**	**Neg**
White	41 (74.5%)	25 (64.1%)	15 (60%)	21 (75%)
Black	7 (12.7%)	6 (15.4%)	3 (12%)	3 (10.7%)
American Indian/Alaska Native	1 (1.8%)	2 (5.1%)	0 (0%)	0 (0%)
Asian	1 (1.8%)	1 (2.6%)	1 (4%)	0 (0%)
Multi-racial	1 (1.8%)	1 (2.6%)	1 (4%)	0 (0%)
Native Hawaiian/Other Pacific Islander	0 (0%)	0 (0%)	1 (4%)	0 (0%)
Non-specified	3 (5.5%)	4 (10.3%)	4 (16%)	4 (14.3%)
Other	1 (1.8%)	0 (0%)	0 (0%)	0 (0%)
Chi-squared p-value = 0.8076				
**Sex**	**As**	**MiL**	**MiH**	**Neg**
Female	6 (10.9%)*	12 (30.8%)	11 (44.0%)	8 (28.6%)
Male	49 (89.1%)*	27 (69.2%)	14 (56.0%)	20 (71.4%)
Chi-squared p-value = 0.0095				

*post-hoc p-value (Bonferroni) = 0.0165.

### IgG, IgM and IgA Specific SARS-CoV-2 Antibodies Are Similarly Induced in Asymptomatic Participants and Participants With Mild Symptoms

**Figure 1:**
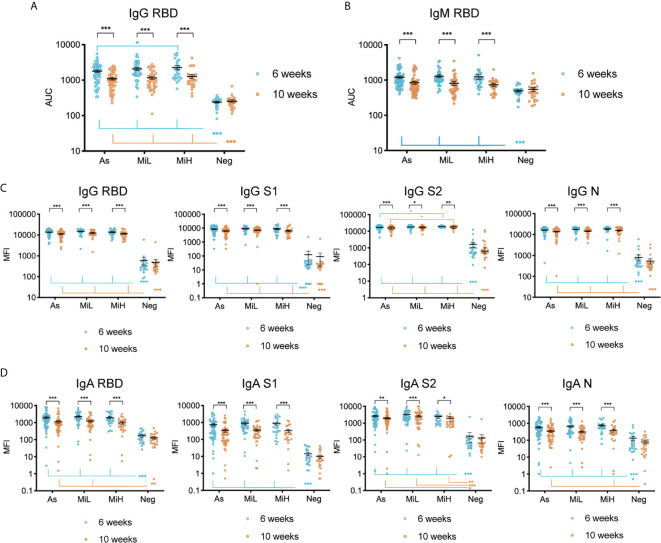
Evaluation of IgG and IgM antibodies against SARS-CoV-2 RBD by ELISA **(A, B)**, and of IgG **(C)** and IgA **(D)** antibodies directed towards SARS-CoV-2 RBD, S1, S2 and N using Luminex xMAP-based multiplex assay in samples from 137 participants (As n=53; MiL n= 36; MiH n= 25, Neg n=23). Mean and SEM are indicated. AUC, Area Under the Curve; MFI, Mean Fluorescence Intensity. Two-way ANOVA test followed by multiple comparisons with Benjamini-Hochberg method (desired FDR 0.05). Adjusted p-values *< 0.05; **< 0.01; ***< 0.001.

We analyzed and compared the levels of IgG and IgM specific to SARS-CoV-2 RBD in young adults with asymptomatic infection and the two different levels of mild symptomatic disease by ELISA, as well as the stability of those antibodies between the 6 and 10 weeks PO. **Figures 1A, B** indicate the AUC for all samples from participants at which data was obtained at both time points (n=137). Representative curves used for AUC calculations including all the dilutions of the assay (OD 492 nm values versus Log10 [dilution factor]) are shown in **Supplementary Figure 1**. We found a significant decrease in both IgG (2.09, 2.17, and 2.46-fold decrease in mean titers in As, MiL and MiH, respectively) and IgM (4.83, 3.19, and 2.11-fold decrease in mean titers in As, MiL and MiH, respectively) antibody titers and AUC values (**Figures 1A, B**) between these two time points in the three groups of participants with antibodies. We found similar levels of IgG for As (mean titer 2753 ± 338 Standard Error of the Mean [SEM]; mean AUC 1790 ± 146 SEM) and MiL (mean titer 3167 ± 529; mean AUC 2077 ± 198) participants at 6 weeks PO, as well as at 10 weeks PO. Interestingly, MiH participants (mean titer 4266 ± 848; mean AUC 2270 ± 310) had slightly higher levels of IgG RBD AUC at 6 weeks PO (adjusted p-value = 0.0475 for titers and 0.0387 for AUC values) than As participants. However, the differences were not significant 4 weeks later (adjusted p-value = 0.6484). No statistically significant differences associated with symptoms were found in IgM RBD antibody levels in SARS-CoV-2 seropositive participants (**Figure 1B**). Additionally, we investigated the association between IgG and IgM RBD AUC with the number of symptoms (**Supplementary Figure 2**), and we did not find a significant correlation at either 6 or 10 weeks PO. Since more male than female participants were in the As group as compared to the other groups (**Table 2**), we next analyzed the effect of sex, in combination with time and being assigned to a specific symptom group, in IgG RBD and IgM RBD responses by performing a three-way ANOVA test (**Supplementary Table**). Sex did not show an effect in IgG RBD AUC values, however, the effect of sex on IgM was significant (p-value= 0.027). No interaction was found between the three variables included in the analysis for IgG or IgM RBD AUC values. A post-hoc analysis comparing IgM RBD AUC values between females and males did not show significant differences between them in any of the groups. However, we found significantly higher levels of IgM in males (adjusted p-value = 0.0166) than in females at 10 weeks PO when the three seropositive groups were combined (As, MiL and MiH, **Supplementary Figure 3**). No significant correlation was identified between number of symptoms and IgG RBD or IgM RBD when females and males were analyzed as separate groups (**Supplementary Figure 2**).

To further characterize the SARS-CoV-2 specific response we evaluated the levels of IgG and IgA specific to different antigens of SARS-CoV-2 using a Luminex xMAP-based multiplex assay (MILLIPLEX^®^). As shown in **Figure 1C**, a robust induction of IgG antibodies against RBD, S1, S2, and N were observed in groups identified as RBD IgG positive by ELISA at both time points, as indicated by significantly higher levels of antibodies than in the Neg group (adjusted p-values < 0.001 in all cases). Similarly to ELISA results, a significant decrease of IgG antibodies was observed for RBD, S1, S2, and N between the 6 and 10 week time points in the three groups of IgG RBD positive participants (**Figure 1C**). Interestingly, we found similar levels of IgG antibodies using this assay for RBD, S1 and N antigens among As, MiL and MiH groups at both time points. However, for IgG S2, we observed slightly higher levels in MiH (mean MFI [mean fluorescence intensity] 19125 ± 574 at 6 weeks PO and 17485 ± 682 at 10 weeks PO, respectively) than in As participants (mean MFI 17073 ± 595 at 6 weeks PO and mean MFI 15491 ± 604 at 10 weeks PO; adjusted p-values = 0.0410 and 0.0479 at 6 and 10 weeks PO, respectively). Luminex analysis of IgA levels against RBD, S1, S2 and N antigens (**Figure 1D**) showed also a strong induction in all the seropositive groups as compared to the Neg group at 6 weeks PO, and highly significant decrease between 6 and 10 weeks PO. IgA against RBD in MiH, S1 in As, MiL and MiH and N in MiL levels at 10 weeks PO were not found to be significantly higher than the Neg group, suggesting a more marked decrease of IgA antibodies in serum than IgG. No differences were observed among the asymptomatic and symptomatic groups in any case for IgA SARS-CoV-2 antibodies. Therefore, we found that SARS-CoV-2 antibody responses are induced to similar levels in participants with asymptomatic and mild COVID-19, with a marginally enhanced IgG antibody production in participants with a higher number of symptoms (MiH) as compared to asymptomatic participants (As).

### SARS-CoV-2 Neutralizing Activity Is Similar Between Participants With Asymptomatic and Mild Infection and Does Not Significantly Decrease From 6 to 10 Weeks PO

**Figure 2:**
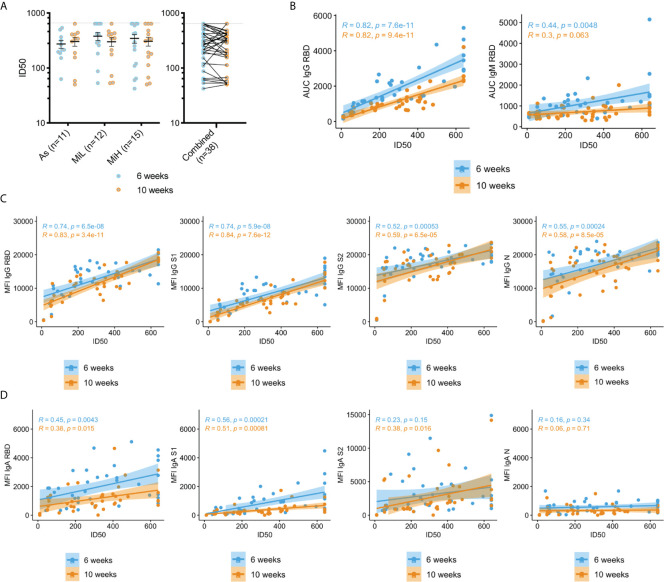
Neutralization activity in sera in As, MiL, MiH or the three groups combined **(A)** and correlation of neutralization ID_50_ values with SARS-CoV-2 specific antibodies **(B–D)**. B) Correlation analysis of RBD IgG and IgM levels calculated by ELISA (AUC values) and neutralization activity (ID_50_). **(C, D)** Correlation analysis of IgG **(C)** and IgA **(D)** antibodies directed towards SARS-CoV-2 RBD, S1, S2 and N using a Luminex xMAP-based multiplex assays (MFI) and neutralization activity (ID_50_). Mean and SEM are indicated. Dash line in A) shows the upper limit of detection. Two-way ANOVA test followed by multiple comparisons with Benjamini-Hochberg method (desired FDR 0.05). ID_50_, half maximal inhibitory dilution. AUC, Area under the curve; MFI, Mean Fluorescence Intensity; *R*, Pearson correlation coefficient; *P*, p-values.

To evaluate the neutralizing activity and its association with symptoms in this cohort, we performed neutralization assays in serum samples from 38 IgG RBD positive individuals (19 female and 19 male; 11 belonging to As [5 female, 6 male], 12 to MiL group [8 female, 4 male] and 15 MiH group [6 female, 9 male]) (**Figure 2A** and **Supplementary Figure 4**) and from two IgG RBD negative individuals. Neutralization assays were performed with a complete SARS-CoV-2 recombinant virus expressing mNeonGreen, which have been previously reported elsewhere (21). Full curves representing percent inhibition for each sera dilution are shown in **Supplementary Figure 4** for the 38 IgG positive participants at 6 and 10 weeks PO. We found that all SARS-CoV-2 IgG positive serum samples included in this analysis possessed neutralizing activity. Notably, no significant decrease was found between 6 and 10 weeks PO, either when analyzed as separate groups (two-way ANOVA time factor: p-value = 0.2113), or when the 38 participants were combined (Wilcoxon matched-pairs signed rank test, two tailed, p-value = 0.1383) (**Figure 2A**), as opposed to the levels of SARS-CoV-2 IgG antibodies (**Figure 1**). This indicates that the protective titer needed for neutralization was maintained during this time despite the drop in total antibody levels. In addition, while a trend towards better neutralizing activity was found in symptomatic participants (MiL mean half maximal inhibitory dilution [ID50] 380 ± 64; MiH mean ID50 344 ± 62 at 6 week PO) than in asymptomatic participants (mean ID50 274 ± 44 SEM at 6 week PO), no statistically significant differences were detected between these groups (Two-way ANOVA symptoms factor: p-value = 0.7997). No significant correlation was found between ID_50_ and the number of symptoms at 6 or 10 weeks PO (**Supplementary Figure 5A**). Samples from two participants of the Neg group were included in the assay and did not show neutralization activity at any of the time points (ID_50_ <10). To evaluate a possible effect of sex in neutralization activity, a three-way ANOVA test, which included group, time and sex as factors, was performed (**Supplementary Table**), and no difference due to sex was identified.

Next, we analyzed the correlation between the neutralization activity and the levels of SARS-CoV-2 specific IgG, IgM and IgA antibodies measured by ELISA or Luminex assays as detailed above (**Figures 2B–D**). High levels of correlation were found between ID_50_ values and IgG RBD (determined either by ELISA or Luminex assays), and IgG S1 (Luminex), which is consistent with the expected inhibitory activity of those antibodies on virus entry. A significant correlation (p-value < 0.001) was also found for IgG antibodies against S2 and N (**Figure 2C**). IgM RBD antibodies were also correlated at the earlier time point with neutralizing activity (p-value = 0.0048), but this correlation was not significant at 10 weeks PO (p-value = 0.063), which is expected given the loss of these antibodies by that time in a high proportion of seropositive participants. The correlation of the neutralization activity with IgA antibodies was overall lower than for IgG, but a strong association was found with RBD and S1 specific antibodies at 6 and 10 weeks PO (p-value < 0.001). Low (S2 IgA at the 10 week time point, p-value = 0.016) or no correlation (p-value > 0.05) was found for IgA against S2 or N proteins. Correlation for IgG and IgM ELISA AUC with ID_50_ values was also evaluated in the individual groups As, MiL and MiH. IgG showed a strong correlation when participants were separated by groups for each specific group (**Supplementary Figure 5B**). IgM at 6 weeks, however, did not yield significant results (**Supplementary Figure 5C**), likely related to a smaller number of participants in each group decreasing the power to detect a difference when compared to the combined analysis.

### Exposure to SARS-CoV-2 Elicits Cross-Reactive Antibody Response to SARS-CoV and MERS-CoV

**Figure 3:**
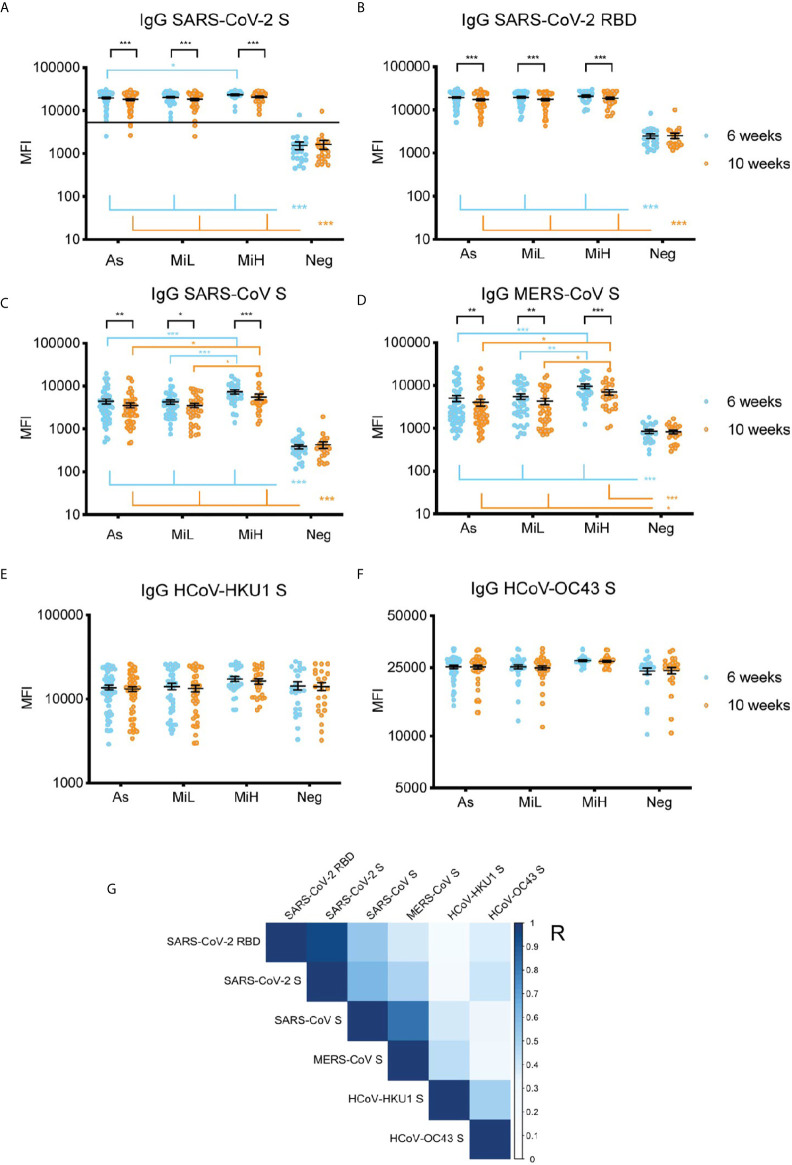
IgG serum reactivity against the RBD and S protein of SARS-CoV-2 **(A, B)** and S from other beta-coronaviruses using an MMIA **(C–F)** and correlation analysis **(G)** in samples from 136 participants (As n=52 [6 female, 46 male]; MiL n= 36 [11 female, 25 male]; MiH n= 25 [11 female, 14 male). Continuous line in **(A)** indicate the threshold of positivity for SARS-CoV-2 S protein (19, 22). Two-way ANOVA test followed by multiple comparisons with Benjamini-Hochberg method (desired FDR 0.05). Adjusted p-values *< 0.05; **< 0.01; ***< 0.001. **(G)** Pearson correlation of IgG reactivity among SARS-CoV-2 RBD, SARS-CoV S, MERS-CoV S, HCoV-HKU1 S and HCoV-OC43 S. R, correlation coefficient.

Next, we investigated the IgG reactivity to the S protein from other betacoronaviruses in the sera of the different groups of SARS-CoV-2 seropositive and seronegative participants using a multiplex microsphere-based immunoassay (MMIA) (18–20). SARS-CoV-2 IgG S and RBD were also included in this assay (**Figures 3A, B**), which showed highly comparable results to the ones obtained by ELISA IgG RBD (**Figure 1A**) or by the Luminex IgG SARS-CoV-2 specific panel (**Figure 1C**). IgG RBD ELISA and MMIA assays showed matching seropositivity results in 98.54% of the samples. The 1.46% discrepancy was due to one participant identified as SARS-CoV-2 IgG RBD positive by ELISA (As group, titer 150) which was identified as negative by MMIA, and one participant in the Neg group (titer 50 by IgG RBD ELISA) who was identified as SARS-CoV-2 positive by MMIA. A significant decrease in MFI was found from 6 to 10 weeks PO in As, MiL and MiH (adjusted p-value < 0.001) groups for both SARS-CoV-2 S and RBD reactive IgG. A slight but significantly higher level in reactivity in MiH as compared to As was found at 6 week PO for SARS-CoV-2 S IgG reactive antibodies (adjusted p-value < 0.0182). All three groups of SARS-CoV-2 IgG positive participants showed a significant development of antibodies against SARS-CoV and MERS-CoV S proteins as compared to the Neg group (**Figures 3C, D**), with similar dynamics to SARS-CoV-2 specific antibodies between the two time points, indicating a high level of IgG cross-reactivity among the S proteins of these zoonotic viruses. Notably, higher levels of antibodies to SARS-CoV and MERS-CoV S proteins were found in the MiH as compared to the As group, which is similar to the results for SARS-CoV-2 S protein (**Figure 3A**) and S2 subunit by Luminex (**Figure 1C**). The differences between As and MiH were more striking for SARS-CoV (adjusted p-value < 0.001 at 6 weeks and < 0.05 at 10 weeks PO) and MERS-CoV (adjusted p-value < 0.001 at 6 weeks and < 0.05 at 10 weeks PO) than for SARS-CoV-2 S. Significant differences were also found between MiL and MiH for SARS-CoV S (adjusted p-value < 0.001 at 6 weeks PO and < 0.05 at 10 weeks PO) and MERS-CoV S (adjusted p-value < 0.01 at 6 weeks PO and p value < 0.05 at 10 weeks PO). The levels of IgG to the S protein of HCoV-HKU1 and HCoV-OC43 were elevated in all groups regardless of exposure to SARS-CoV-2 and did not show significant change over the two time points (**Figures 3E, F**). We next evaluated the effect of sex on IgG levels specific for SARS-CoV-2 RBD, as well as for S protein of SARS-CoV-2, SARS-CoV, and MERS-CoV S, from the MMIA assay. Interestingly, significantly higher IgG levels were found in females than males in the asymptomatic group (**Supplementary Figure 6**) at 6 weeks for SARS-CoV-2 RBD and S, SARS-CoV S, and MERS-CoV S. In the case of SARS-CoV and MERS-CoV S differences between male and female were also significant at 10 weeks PO in the asymptomatic group. SARS-CoV also showed sex-specific differences in MiL group. Therefore, these data suggest that asymptomatic females might have elevated IgG antibody responses when compared to males. However, due to the low number of asymptomatic females in this cohort (n=6) and weak significance levels (SARS-CoV-2 RBD 6 weeks p-value = 0.029, SARS-CoV-2 S 6 weeks p-value = 0.023), further investigation would be required in a separate cohort to evaluate this hypothesis.

A correlation analysis showed a significant association among the IgG levels against S proteins from the different betacoronaviruses included in this assay (**Figure 3G**). IgG reactivity to S and RBD showed a very strong correlation (Pearson coefficient [R]=0.96, p-value <0.001), as expected. Across different viruses, the highest correlations with SARS-CoV-2 S protein reactive antibodies were found for SARS-CoV S protein (R=0.63, p-value <0.001), and MERS-CoV S protein (R=0.49, p-value <0.001). SARS-CoV-2 IgG antibodies positively correlated also with HCoV-HKU1 (R=0.25, p-value<0.001), and HCoV-OC43 (R=0.40; p-value <0.001) S proteins suggesting the elicitation of IgG antibodies directed to regions of the S protein conserved between SARS-CoV-2 and HCoV after SARS-CoV-2 exposure. Of note, the identity between the S protein of these coronaviruses with SARS-CoV-2 (Uniprot ID P0DTC2) is about 76% for SARS-CoV (Uniprot ID P59594), which showed the highest correlation in our analysis, 29% for MERS-CoV (Uniprot ID K9N5Q8), 28% for HCoV-OC43 (Uniprot ID P36334) and 27% for HCoV-HKU1 (Uniprot ID Q0ZME7) (Alignments performed at www.uniprot.org [Clustal Omega program]).

Overall, we found that exposure to SARS-CoV-2 resulted in *de novo* polyclonal IgG responses that cross-react with the zoonotic coronaviruses SARS-CoV and MERS-CoV, and that this response is correlated with the reactivity to the S protein from four related zoonotic and seasonal betacoronaviruses.

## Discussion

In this study, we have investigated the immune responses to SARS-CoV-2 virus in healthy young adults that were exposed to SARS-CoV-2 virus during an outbreak at Marine Corps Recruit Depot Parris Island (MCRDPI) in the spring of 2020. At this early stage of the pandemic, SARS-CoV-2 virus testing was a limited resource, and therefore only a few participants in this study were PCR confirmed as SARS-CoV-2 positive. Therefore, many of the participants that were isolated from other training recruits were suspected cases based on a clinical diagnosis or close contact with confirmed or suspected cases. To characterize this outbreak, we utilized serology as indirect evidence of SARS-CoV-2 virus exposure as well as medical records collected during the outbreak for evaluation of disease severity in a group of 147 participants that were identified as exposed or potentially exposed.

Multiple studies have previously analyzed the association of COVID-19 severity and antibody responses, and found that asymptomatic individuals tend to generate lower magnitude of antibody responses than symptomatic individuals (10, 11, 23). However, these studies include patients in different age populations and worse clinical severity, in some cases requiring hospitalization, than participants in this study. We found that 81% of participants had RBD IgG antibodies in sera 6 weeks PO. Of those, 54% reported symptoms associated with mild disease, while the rest were asymptomatic. Given the heterogenicity in the number and type of symptoms reported, we established two levels in the group of seropositive participants that presented with symptoms (MiL and MiH, **Table 1**). Interestingly, we observed similar levels of SARS-CoV-2 IgG, IgM or IgA antibody responses elicited in asymptomatic participants and in the two outpatient groups of participants who experienced mild COVID-19. Slightly higher antibody levels (RBD-reactive IgG by ELISA, S2-reactive IgG by Luminex, and S protein-reactive IgG by MMIA) were detected in the participants with higher number of symptoms (MiH group). This indicates that, overall, in an age-controlled, young healthy cohort, differences in the magnitude of the polyconal antibody response could not be observed between asymptomatic participants and symptomatic outpatients stratified based on mild COVID-19 symptoms. Similarly, the presence of neutralization antibodies in sera was no different between symptomatic and asymptomatic participants.

We found a significant decrease of levels of antibodies in sera during the subsequent weeks after infection, however most of the seropositive participants maintained detectable IgG antibody levels at 10 weeks PO. These circulating antibody dynamics are consistent with previous studies (5, 7, 24–26). The peak in serum antibodies and the subsequent decline is likely explained by the dynamics of circulating short-lived plasma cells or plasmablasts, which peak in blood at 7 days after infection (27). Once in circulation, IgG molecules have a half-life of approximately 3 weeks (28). Long-lived plasma cells (LLPC), which are generated through germinal center reactions and then traffic to bone marrow, account for long term persistence of circulating IgG later after infection (29, 30). Affinity maturation drives selection of LLPC, which increases possibility of better neutralizing properties of the antibodies produced at later times after infection (31), while the plasmablasts generated in the early extrafollicular response experiment limited somatic hypermutation, and the affinity of the resulting antibodies might be moderate (32, 33). Interestingly, in our study, neutralizing activity in serum was maintained during the study period, despite the decrease in IgG, IgA and IgM antibody levels. The dynamics of the source and quality of the antibody response might account for the higher stability of the neutralizing response as compared to antibody levels observed in this study. In agreement with this hypothesis, correlation between neutralization and RBD and S1 IgG levels, measured by Luminex assay, showed slightly higher coefficients and lower p-values at 10 weeks PO than at 6 weeks PO (**Figure 2C**). Similarly, Garritsen et al. found higher stability of sera neutralizing antibody as compared to S1 IgG levels in two tests performed separated 46 – 96 days, at various times after infection (34). However, other studies have found a decline in neutralizing antibodies at times after exposure that were similar to this study (10, 24) which could be attributed to different study populations and clinical differences.

Analysis of the reactivity with related betacoronaviruses showed high levels of IgG antibodies to the S protein of SARS-CoV and MERS-CoV in SARS-CoV-2 seropositive participants, as well as a significant correlation between SARS-CoV-2 S IgG levels and SARS-CoV, MERS-CoV, HCoV-HKU1 and HCoV-OC43. Sera samples from SARS-CoV had been previously shown to react against SARS-CoV-2 S protein (35). In this study, it is not possible to demonstrate whether SARS-CoV-2 infection results in the boosting of HCoV-HKU1 or HCoV-OC43 antibodies, since we did not have access to serum baseline samples (before SARS-CoV-2 exposure) as participant enrollment and sample collection occurred after the outbreak. However, previous studies have found a boosting effect of antibodies to seasonal coronaviruses after SARS-CoV-2 infection (36, 37). Antibodies specific to SARS-CoV and MERS-CoV were also found in SARS-CoV-2 IgG positive individuals in another study (19, 22) using the same MMIA assay utilized here. The SARS-CoV-2 S2 subunit has been demonstrated to stimulate pre-existing HCoV memory B cells (38), and since S2 is the region with highest conservation across coronaviruses this is most likely the region targeted by cross-reactive antibodies (39).

This study has several limitations because of the characteristics of the cohort. First, there is a narrow range of ages (18–26), which implies that these findings cannot be generalized to older adults or children. Also, there is a high representation of White participants, and therefore these findings might not apply to other races. In addition, there is a low representation of females in the cohort, which provides reduced power to confidently address sex-specific differences. Finally, Marine recruits are presumable more active than the overall young population, and were prescreened for physical and mental conditions incompatible with military service. However, our study provides a better understanding of the humoral immune responses to SARS-CoV-2 in healthy and physically active young adults, eliminating concerns of the effect of age as a confounding factor of immune responses, as strong associations between age, severity and antibody responses have been previously described (25). Overall, we found that young adults with asymptomatic and mild COVID-19 develop similar humoral antibody responses to SARS-CoV-2. While a statistically significant decrease of the levels of IgG is found in all SARS-CoV-2 IgG positive participants from 6 to 10 weeks PO, the magnitude of this decrease is low, and 97.3% of the individuals that were positive at 6 weeks PO and were also tested at 10 weeks PO maintained detectable antibodies. In addition, no significant changes in the levels of neutralizing antibodies were observed in this time frame. Finally, we observed a significant correlation between the presence of SARS-CoV-2 specific antibodies and four related medically-relevant betacoronaviruses, indicating that SARS-CoV-2 exposure elicits induction of antibodies against epitopes conserved among them, which may have broad implications for the development of pan-coronavirus vaccines.

## Data Availability Statement

The raw data supporting the conclusions of this article will be made available by the authors, without undue reservation.

## Ethics Statement

The studies involving human participants were reviewed and approved by Naval Medical Research Center (NMRC) institutional review board (IRB). The patients/participants provided their written informed consent to participate in this study.

## Author Contributions

IR: performed and supervised serological assays, analyzed data, prepared figures, wrote the manuscript. CG: contributed to data collection and data analysis, wrote the manuscript. AS-S, ECS, SP, MM, KH, CAP, NM, and SV performed serological assays. DLW, BLP, JR, MPS, SL, RL: contributed to sample and data collection. M-CG, VDN and MST provided project administration and coordination. GRS, WM: contributed to data analysis. MC, EDL, CCB, AB: supervised data generation and data analysis. SCS and AGL: supervised data collection, data generation, data analysis and provided overall leadership to the investigation. All authors contributed to the article and approved the submitted version.

## Funding

This work received funding from the Defense Health Agency through the Naval Medical Research Center (9700130) and from the Defense Advanced Research Projects Agency (contract number N6600119C4022).

## Disclaimer

The views expressed in the article are those of the authors and do not necessarily express the official policy and position of the US Navy, the Department of Defense, Uniformed Services University, the US government or the institutions affiliated with the authors.

## Conflict of Interest

The authors declare that the research was conducted in the absence of any commercial or financial relationships that could be construed as a potential conflict of interest.​​​​​​​​​​​​​​​​​​​​
