# Supplementary material for: Antibody Responses to SARS-CoV-2 Following an Outbreak Among Marine Recruits With Asymptomatic or Mild Infection
Source: Front Immunol. 2021 Jun 9;12:681586. doi: 10.3389/fimmu.2021.681586 (PMC8220197; doi:10.3389/fimmu.2021.681586)

Supplementary Material

1. Supplementary Table and Figures

Supplementary Table. Three-way ANOVA analysis of IgG RBD and IgM RBD Area Under the Curve (AUC) and ID_{50}. Table shows p-values for the effect of groups, time and sex in IgG, IgM RBD AUC values and ID_{50}, as well as the interaction among these variables. For IgG and IgM, group variable includes: As (53 participants [6 female, 47 male], n=106), MiL (36 participants [11 female, 25 male], n=72), MiH (25 participants [11 female, 14 male]; n=50), and Neg (23 participants [4 female, 19 male]; n=46); Time variable includes: 6 weeks (n=137) and 10 weeks (n=137); Sex variable includes: Female (32 participants, n= 64) and Male (105 participants, n= 210). For ID_{50}, group variable includes: As (11 participants [5 female, 6 male], n=22), MiL (12 participants [8 female, 4 male], n=24), MiH (15 participants [6 female, 9 male]; n=30); Time variable includes: 6 weeks (n=38) and 10 weeks (n=38); Sex variable includes: Female (19 participants, n=38); Male (19 participants, n=38). Significance: * p-value <0.05, ** p-value <0.01, p-value <0.001 ***.

	Variable effect	p-value	Significance
IgG RBD	Group	5.25E-15	***
	Time	3.40E-09	***
	Sex	0.315	
	Group:Time	0.056	
	Group:Sex	0.428	
	Time:Sex	0.199	
	Group:Time:Sex	0.753	
IgM RBD	Group	8.28E-06	***
	Time	1.32E-05	***
	Sex	0.027	*
	Group:Time	0.138	
	Group:Sex	0.404	
	Time:Sex	0.945	
	Group:Time:Sex	0.645	
ID_{50}	Group	0.543	
	Time	0.582	
	Sex	0.536	
	Group:Time	0.712	
	Group:Sex	0.094	
	Time:Sex	0.721	
	Group:Time:Sex	0.756	

Supplementary Figure 1. Examples of IgG and IgM ELISA titration curves for Area Under the Curve (AUC) calculations at 6 and 10 weeks PO. Optical density (OD) at 492 nm versus Log10 (dilution factor) is represented in every plot. Participant identifiers are shown in the top right corners of the plots. A) Three participants from As group; B) Three participants from MiL group; C) Three participants from MiH group.

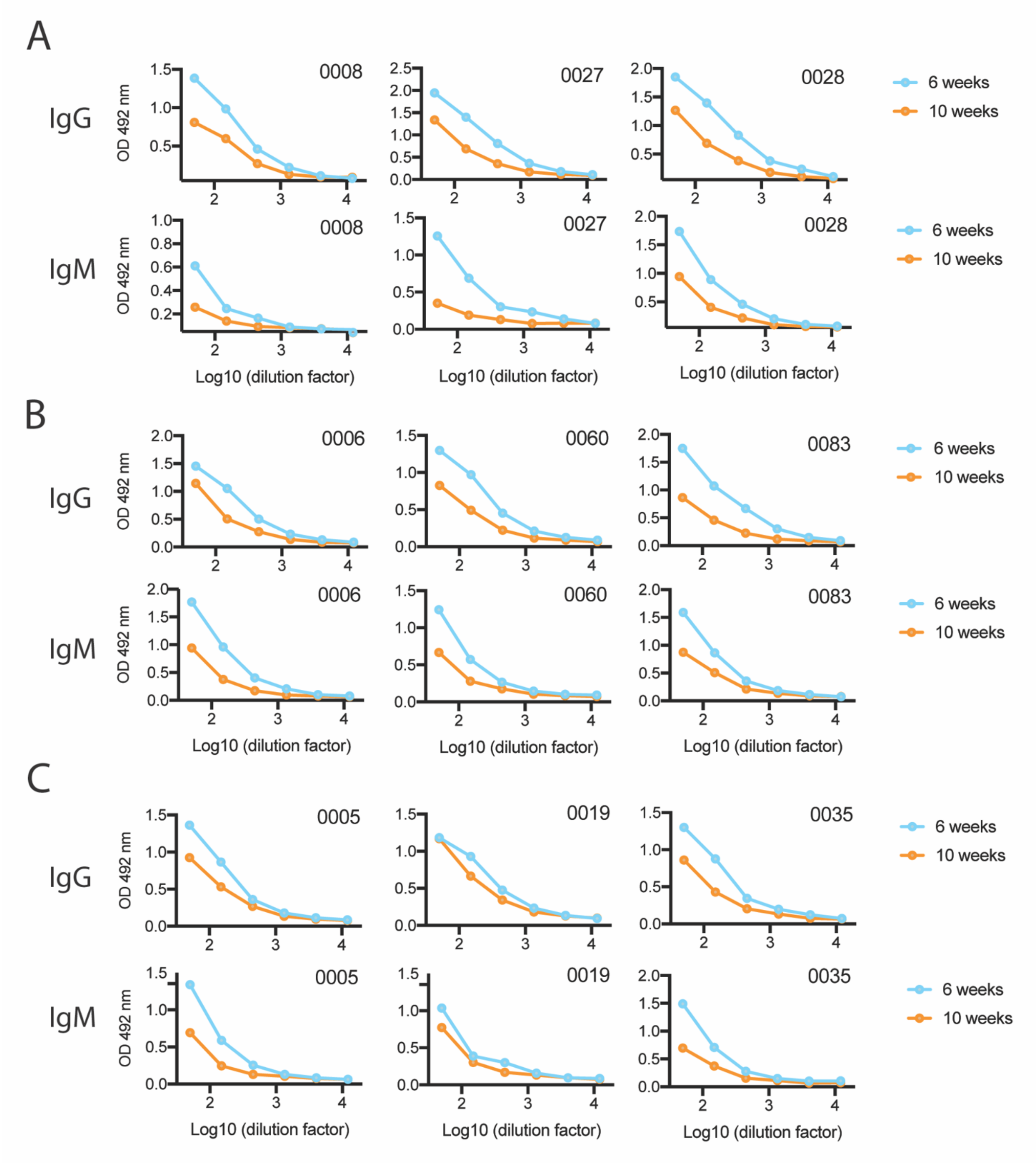

Supplementary Figure 2. Correlation analysis of IgG and IgM RBD Area Under the Curve (AUC) with number of symptoms in all 137 participants combined or stratified by sex.

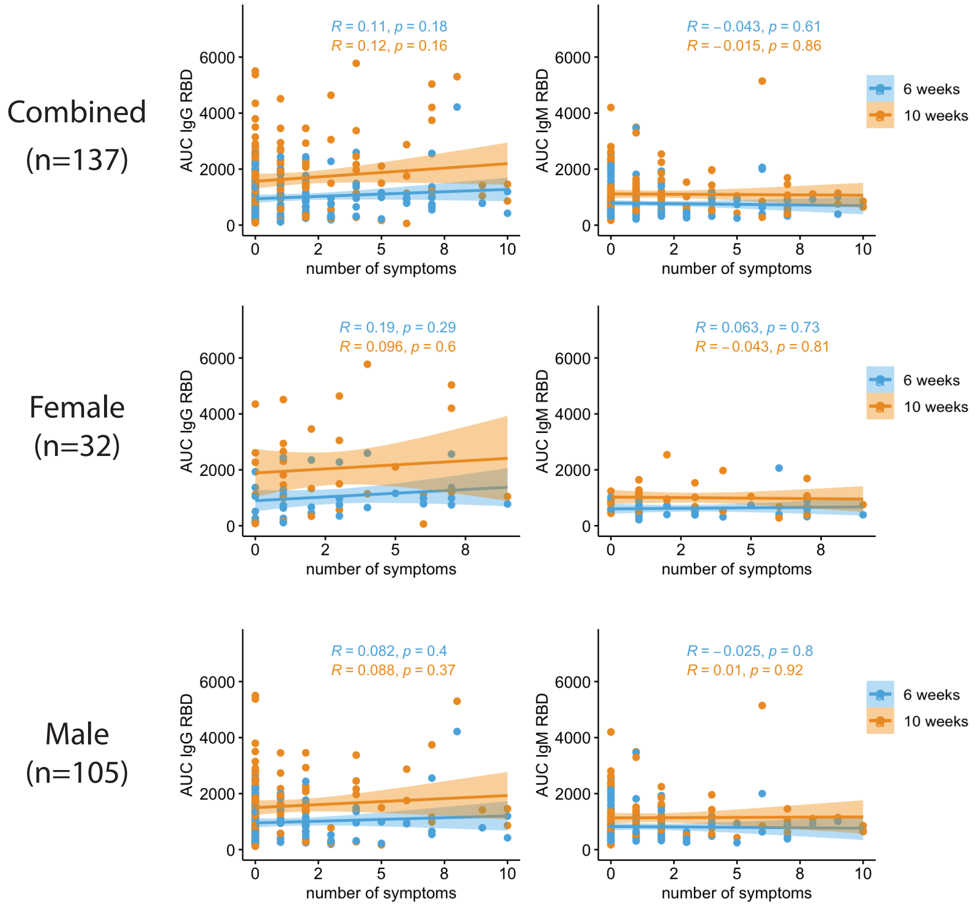

Supplementary Figure 3. Comparison of IgM RBD of Area Under the Curve (AUC) levels in seropositive (Pos: As, MiL and MiH groups combined) and seronegative (Neg) female and male participants.

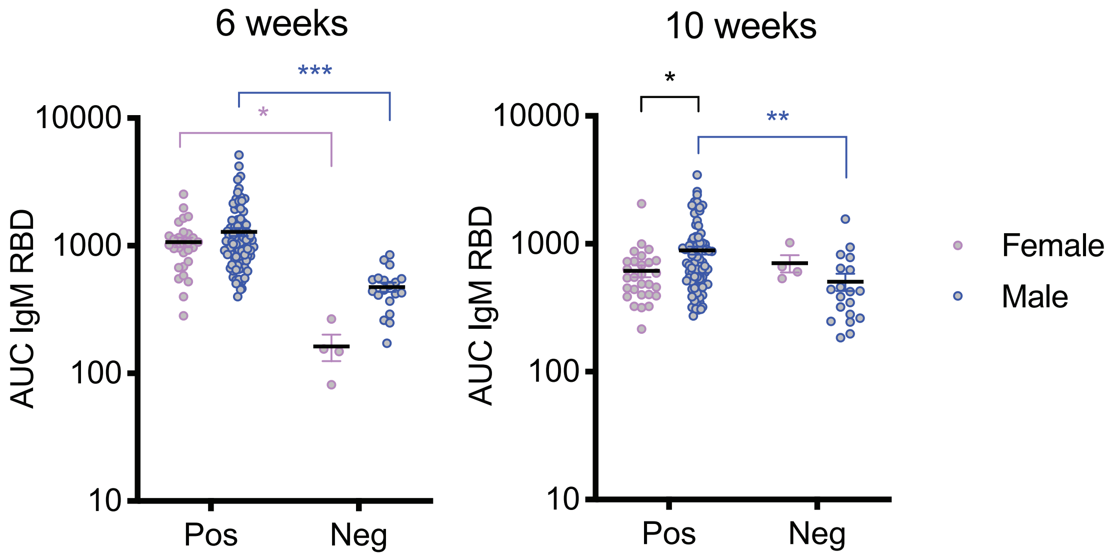

Supplementary Figure 4. Full curves for quantification of ID_{50} of neutralization assays for participants in As (A), MiL(B) and MiH (C) groups. Percent inhibition (calculated as percent of virus infection in the absence of sera) versus Log10 (dilution factor) are represented, and 4-parameter logistic regression method was used to calculate the serum dilution at which 50% of virus was neutralized (half-maximal inhibitory serum dilution; ID_{50}). Participant identifiers are shown in the top right corners of the plots. Data for 6 and 10 weeks are plotted for each participant for comparison. Calculated ID_{50} values are shown for every participant.

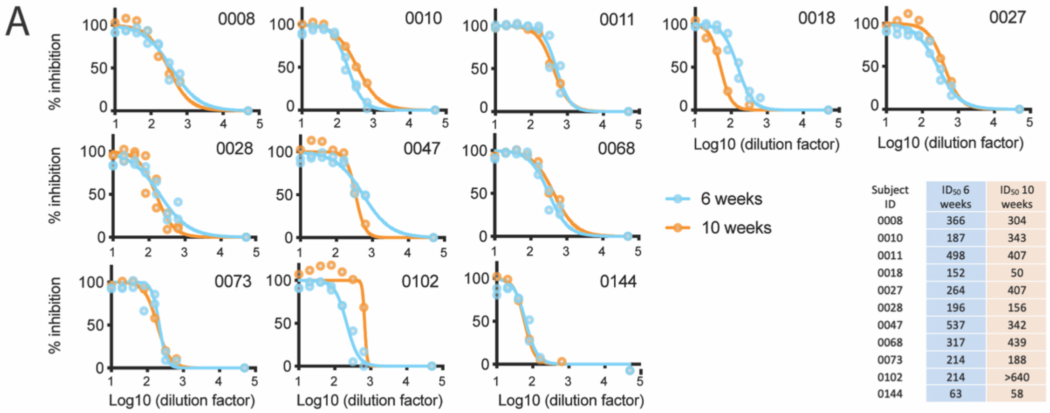

Subject ID	ID_{50} 6 weeks	ID_{50} 10 weeks
0008	366	304
0010	187	343
0011	498	407
0018	152	50
0027	264	407
0028	196	156
0047	537	342
0068	317	439
0073	214	188
0102	214	>640
0144	63	58

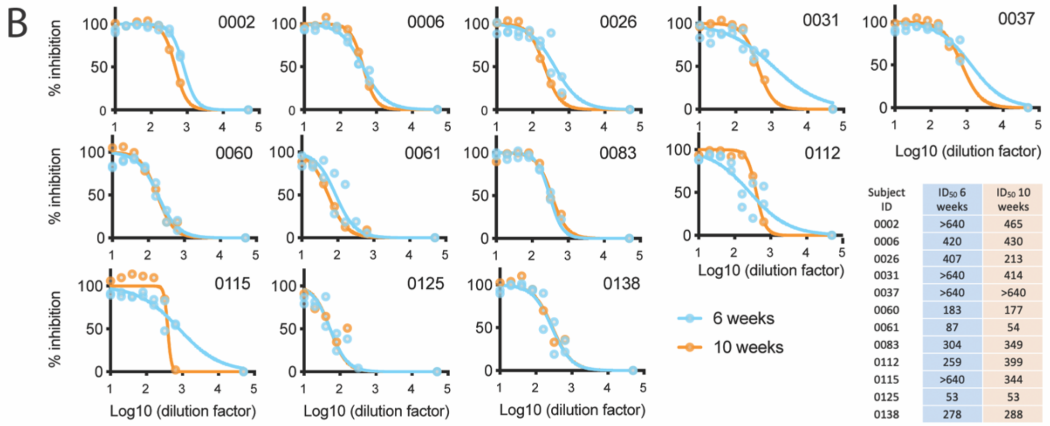

Subject ID	ID_{50} 6 weeks	ID_{50} 10 weeks
0002	>640	465
0006	420	430
0026	407	213
0031	>640	414
0037	>640	>640
0060	183	177
0061	87	54
0083	304	349
0112	259	399
0115	>640	344
0125	53	53
0138	278	288

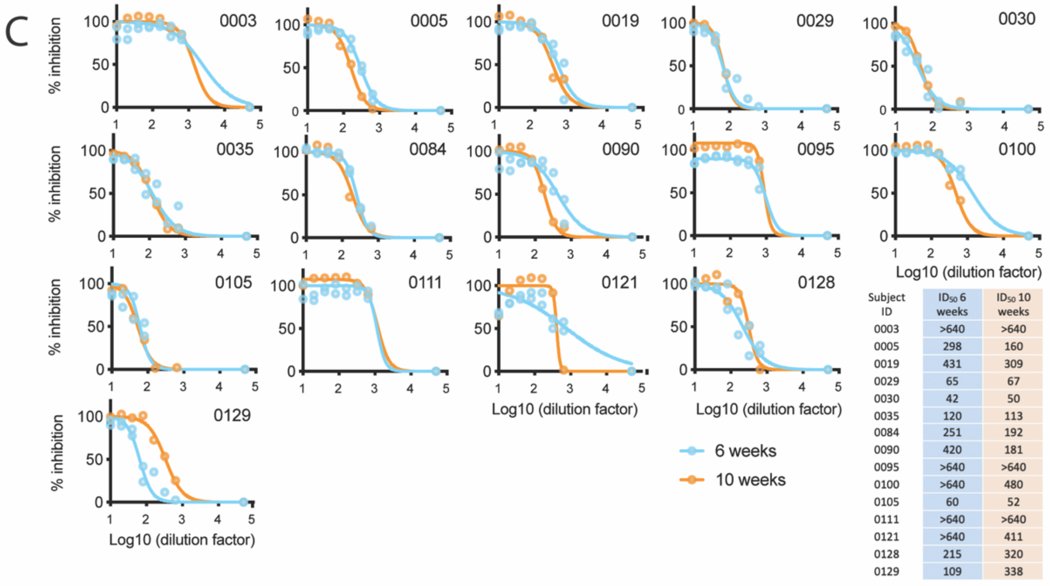

Subject ID	ID_{50} 6 weeks	ID_{50} 10 weeks
0003	>640	>640
0005	298	160
0019	431	309
0029	65	67
0030	42	50
0035	120	113
0084	251	192
0090	420	181
0095	>640	>640
0100	>640	480
0105	60	52
0111	>640	>640
0121	>640	411
0128	215	320
0129	109	338

Supplementary Figure 5. Correlation analysis of ID_{50} values with number of symptoms in 38 participants combined or stratified by sex (A) and with IgG (B) and IgM (C) RBD Area Under the Curve (AUC) values in As (n=11), MiL (n=12) and MiH (n=15) groups.

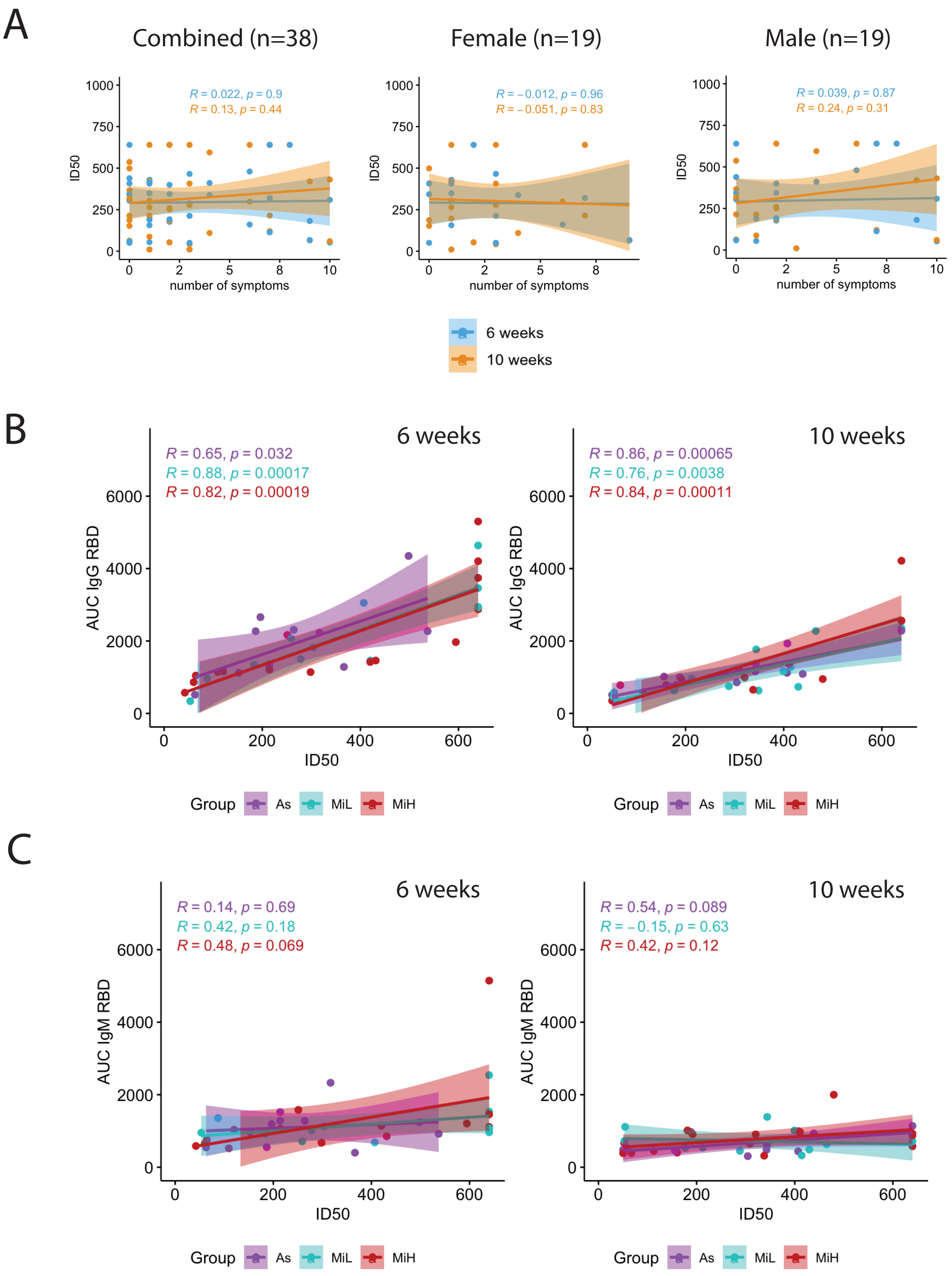

Supplementary Figure 6. Differences between females (F) and males (M) in IgG specific for SARS-CoV-2 RBD, and S from SARS-CoV-2, SARS-CoV and MERS-CoV, generated using a multiplex microsphere-based immunoassay (MMIA). MFI: Mean of fluorescence intensity. Adjusted p-values *<0.05; **<0.01; ***<0.001.

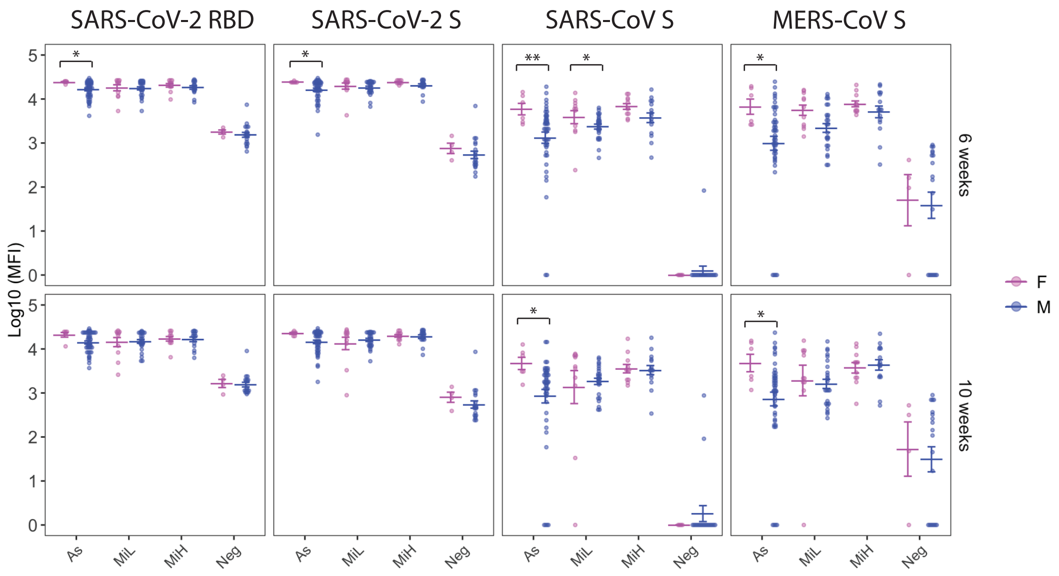

Supplement: Supplementary file 1 [file DataSheet_1.pdf]
